# Assessment of Additional Medical Costs Among Older Adults in Japan With a History of Childhood Maltreatment

**DOI:** 10.1001/jamanetworkopen.2019.18681

**Published:** 2020-01-08

**Authors:** Aya Isumi, Takeo Fujiwara, Hirotaka Kato, Taishi Tsuji, Daisuke Takagi, Naoki Kondo, Katsunori Kondo

**Affiliations:** 1 Department of Global Health Promotion, Tokyo Medical and Dental University, Tokyo, Japan; 2 Japan Society of the Promotion of Science, Tokyo, Japan; 3 Keio University Graduate School of Business Administration, Tokyo, Japan; 4 Center for Preventive Medical Sciences, Chiba University, Chiba, Japan; 5 Department of Health and Social Behavior and Department of Health Sociology and Health Education, The University of Tokyo, Tokyo, Japan; 6 Center for Gerontology and Social Science, National Center for Geriatrics and Gerontology, Obu, Japan

## Abstract

**Importance:**

Childhood maltreatment can have significant consequences on health through the life course, but its association with health care costs in later life is not widely known.

**Objective:**

To assess whether a history of childhood maltreatment is associated with additional medical costs among older adults in Japan.

**Design, Setting, and Participants:**

This population-based cross-sectional study used data from the Japan Gerontological Evaluation Study, 2013, linked with national health insurance claims data from April 2012 to March 2014 for 1 municipality that participated in the Japan Gerontological Evaluation Study, 2013. The municipality had more than 1.5 million residents, and 978 independent individuals aged 65 to 75 years were included in the analysis. Data were analyzed from October 2017 to February 2019.

**Exposures:**

Childhood maltreatment, including physical abuse, emotional neglect, emotional abuse, and witnessing intimate partner violence.

**Main Outcomes and Measures:**

Mean annual medical costs between April 2012 and March 2013 and between April 2013 and March 2014.

**Results:**

Among 978 independent older adults (mean [SD] age, 70.6 [2.9] years; 426 [43.6%] men), 44 (4.5%) witnessed intimate partner violence, 19 (1.9%) were physically abused, 104 (10.6%) were emotionally neglected, and 56 (5.7%) were emotionally abused in childhood. In total, 176 older adults (18.0%) experienced at least 1 type of childhood maltreatment. Mean annual medical costs of those who experienced any childhood maltreatment were significantly higher than of those who did not (difference, ¥136 456 [US$1255]; 95% CI, ¥38 155-¥234 757 [US$351-US$2160]; *P* = .007). Those who experienced emotional neglect incurred more mean medical costs than those who did not (difference, ¥161 400 [US$1485]; 95% CI, ¥42 779-¥280 021 [US$394-US$2576]; *P* = .008). The association of any childhood maltreatment with medical costs remained significant after controlling for age and sex (average marginal effect, ¥116 098 [US$1068]; SE, ¥53 620 [US$493]; 95% CI, ¥11 004-¥221 192 [US$101-US$2035]; *P* = .03). The estimated additional costs associated with childhood maltreatment would be more than ¥333 billion (US$3.1 billion) per year nationwide.

**Conclusions and Relevance:**

In this study, childhood maltreatment was associated with additional medical costs among older adults living in Japan. This finding underlines the importance of primary and secondary prevention of child maltreatment.

## Key Points

**Question:**

Are medical costs higher among older adults with a history of childhood maltreatment than among those without?

**Findings:**

In this cross-sectional study of 978 adults, using national health insurance claims data in Japan, mean annual medical costs among those who experienced any childhood maltreatment were significantly higher than costs among those who did not, even after adjusting for age and sex.

**Meaning:**

In this study, childhood maltreatment was associated with additional medical costs among older adults living in Japan, which highlights the importance of primary and secondary prevention of child maltreatment.

## Teaser

This cross-sectional study assesses whether a history of childhood maltreatment is associated with additional medical costs among older adults in Japan.

## Introduction

Childhood maltreatment can have long-term consequences on health through the life course^1,2,3,4,5^ and is associated with a higher risk of mental^3,6,7,8,9,10,11,12,13,14^ and physical^3,15,16,17,18,19,20,21,22,23,24^ health issues in adulthood. Furthermore, recent research using a cohort study of independent adults older than 65 years in Japan^25,26^ has shown that childhood maltreatment can lead to deteriorating health in late adulthood, including greater higher-level functional limitations and greater risk of diseases such as diabetes and hypertension.

Therefore, experiencing childhood maltreatment may lead to additional health care costs in later life.^27,28^ Previous studies in the United States using health care system data^29,30^ have shown that childhood sexual abuse and/or physical abuse were associated with an increase in annual health care costs among women aged 18 to 65 years after controlling for demographic characteristics. Similar results were obtained in a Canadian study using ambulatory self-reported health care costs,^31^ although the amount of annual health care costs was smaller than in studies that used health care system data. Notably, these previous studies exclusively targeted women who were younger than 65 years. To our knowledge, the long-term association of childhood maltreatment with health care costs among individuals aged 65 years or older, including men, has not been examined. Moreover, no previous studies on health care costs have included the associations of childhood emotional abuse and neglect with health care costs.

In Japan, the social costs of childhood maltreatment in 2012 were reported to be ¥1.6 trillion (US$14.7 billion), including direct and indirect costs.^32^ However, the actual medical costs of those who experienced childhood maltreatment are still unknown because medical costs were estimated indirectly in the study. As the Japanese population is aging, elucidating the association of childhood maltreatment with health care costs among older adults is critical.

This study aimed to assess additional medical costs associated with childhood maltreatment, using the database of health insurance claims that is linked with data from the Japan Gerontological Evaluation Study (JAGES), 2013, a population-based cohort of independent adults aged 65 years or older across Japan. We also calculated additional annual medical costs for different types of childhood maltreatment (ie, witnessing intimate partner violence [IPV] and experiencing physical abuse, emotional neglect, and emotional abuse).

## Methods

### Data and Sample

Our sample was based on participants of JAGES 2013, which was designed to investigate the social determinants of health among noninstitutionalized, functionally independent individuals aged 65 years or older. Started in 2003, JAGES is the largest cohort study of older adults in Japan, with samples from 30 municipalities in 15 prefectures.

Data from JAGES 2013 were linked with the database of national health insurance claims for a city with more than 1.5 million residents, of whom 7257 were aged 75 years or younger in 2013 (ie, those who were ≤74 years in fiscal year [FY] 2012 [ie, April 2012 to March 2013]). Individuals with employer-sponsored health insurance or those who receive public assistance are not eligible for national health insurance. The database of health well care visits for those with national health insurance was used to distinguish between individuals who did not use national health insurance during FY2012 and FY2013 (66 individuals) and individuals who may not have been eligible for national health insurance. Those who may not have been eligible for national health insurance, including those who have employer-sponsored health insurance and those who receive public assistance, were excluded from the sample (2343 in FY2012; 2325 in FY2013). Medical and pharmacy claims of 5155 individuals were available for either FY2012 or FY2013. A sample of 978 individuals was used for this analysis because a randomly selected fifth of the JAGES 2013 sample was asked questions regarding adverse childhood experiences. An additional 34 participants who did not answer all 4 questions regarding childhood maltreatment were also excluded (Figure). This study was approved by the institutional review board at Chiba University in Chiba, Japan, and informed consent was waived because this study used secondary data. This study followed the Strengthening the Reporting of Observational Studies in Epidemiology (STROBE) reporting guideline.

**Figure.**
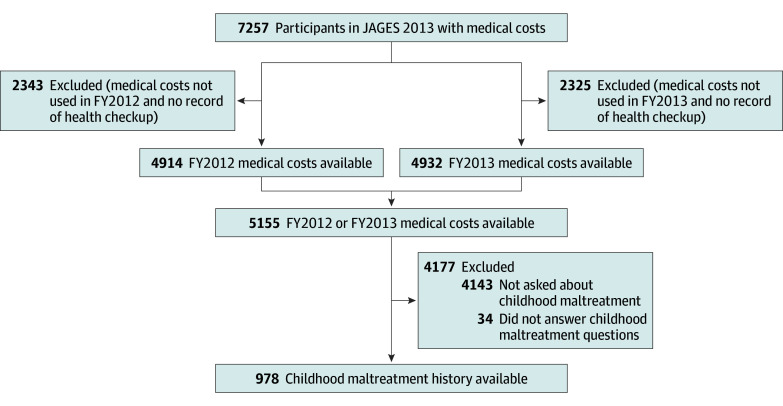
Flowchart of Study Participants Fiscal year (FY) was defined as April to March. JAGES indicates the Japan Gerontological Evaluation Study.

### Measurement

#### Medical Costs

Mean annual medical costs, including publicly funded health care costs, for FY2012 and FY2013 were calculated from medical fee points (1 point indicates ¥10, ie, the unit of cost is ¥10), which were recorded in the database of national health insurance claims for city residents. These medical costs included outpatient, inpatient, and pharmaceutical costs but not dental care costs. When medical costs were available for both FY2012 and FY2013, the mean annual medical cost was used, and if only 1 FY was available, the available annual data were used as the mean annual cost for the 2 FYs.

#### History of Childhood Maltreatment

History of maltreatment before the age of 18 years was assessed as part of the adverse childhood experiences questions in the JAGES 2013 survey, based on previous studies^1^ and modified to suit to older adults living in Japan.^25^ Questions regarding the 4 following types of childhood maltreatment were asked: (1) witnessing IPV, (2) experiencing physical abuse, (3) experiencing emotional neglect, and (4) experiencing emotional abuse. A participant was classified as having witnessed IPV if they answered yes to the statement, “Your father was violent with your mother.” Witnessing IPV was included as a type of childhood maltreatment in this study because IPV in front of children has been defined as child maltreatment in Japan since the Child Abuse Prevention Act was passed in 2004. A participant was classified as having experienced physical abuse if they answered yes to the statement, “(You) were hit hard by your mother/father, causing an injury.” A participant was classified as having experienced emotional neglect if they answered no to the statement “(You) felt loved by your parents.” A participant was classified as having experienced emotional abuse if they answered yes to the statement “(You) were told hurtful things or were insulted by your mother/father.” We aggregated the responses for any type of child maltreatment. In addition, the total number of maltreatment types experienced in childhood was used to assess the cumulative association of multiple types of child maltreatment with health care costs.

#### Covariates

Age and sex of older adults measured in the JAGES 2013 survey were used. Self-reported disease history was measured in JAGES 2013 using the following response items: high blood pressure; stroke; heart disease; diabetes; hyperlipidemia (ie, lipid abnormality); respiratory disease; gastrointestinal, liver, or gallbladder disease; kidney or prostate gland disease; musculoskeletal disease; traumatic injury; cancer; blood or immune system disease; depression; dementia; Parkinson disease; eye disease; ear disease; and other diseases.

### Statistical Analysis

Mean annual medical costs were calculated and compared using * t *tests between those who experienced any type of childhood maltreatment and those who experienced none. Considering that the distribution of medical costs was highly skewed (eFigure in the Supplement), a generalized linear model (GLM) was used to examine the association of childhood maltreatment history with medical costs. Model 1 was adjusted for age and sex because these characteristics were assumed to be preexposure variables that would be associated with a history of childhood maltreatment and medical costs in late adulthood (model 1).^33,34^ We used a χ^2^ test to determine which self-reported diseases were associated with a history of childhood maltreatment (eTable 1 in the Supplement). These diseases were added in model 2 to explore whether medical history mediated the association of childhood maltreatment and additional medical costs. In both GLMs, log-link function and gamma distribution for the mean-variance association were specified according to the Box-Cox test and modified Park test.^35^ The marginal effects of the exposure variable and covariates in Japanese yen were computed from the GLMs.

We conducted *t* tests and GLMs for each type of childhood maltreatment and the total number of childhood maltreatment experiences. Model 2 in the GLM was only performed on types of childhood maltreatment that were found to be significant in *t* tests. The GLMs were conducted with complete data (ie, missing values for each type of childhood maltreatment and covariates were excluded); the largest percentage of missing values was 1.6%, for emotional neglect. Statistical significance was set at *P* < .05, and all tests were 2-tailed. All analyses were conducted using Stata version 14.1 (StataCorp) from October 2017 to February 2019.

## Results

Table 1 summarizes the characteristics, health conditions, and prevalence of childhood maltreatment among the total sample of 978 participants and compares them between 176 participants (18.0%) with at least 1 type of childhood maltreatment and 802 participants (82.0%) without. Overall, 44 participants (4.5%) witnessed IPV, 19 (1.9%) were physically abused, 104 (10.6%) were emotionally neglected, and 56 (5.7%) were emotionally abused in their childhood. The mean (SD) age of the sample was 70.6 (2.9) years, and men accounted for slightly less than half of the sample (426 [43.6%]). Those who experienced any childhood maltreatment were significantly younger (mean [SD] age, 70.3 [2.8] years vs 70.7 [2.9] years; *P* = .046) and less educated (≥13 years of education, 41 [23.3%] vs 266 [33.2%]; *P* = .01), had lower self-rated health (poor health, 5 [2.8%] vs 10 [1.2%]; *P* < .001), and had a higher prevalence of kidney or prostate gland disease (19 [10.8%] vs 38 [4.7%]; *P* = .002) and musculoskeletal disease (25 [14.2%] vs 74 [9.2%]; *P* = .047) than those who did not. Participants who experienced physical abuse had a higher prevalence of stroke and cancer than those who did not (stroke, 3 [15.8%] vs 27 [2.8%]; *P* = .001; cancer, 3 [15.8%] vs 40 [4.2%]; *P* = .02) (eTable 1 in the Supplement). Participants who experienced emotional neglect had a higher prevalence of kidney or prostate disease than those who did not (11 [10.6%] vs 43 [5.0%]; *P* = .02) (eTable 1 in the Supplement). A total of 36 participants (3.7%) reported experiencing at least 2 types of childhood maltreatment.

**Table 1.** Characteristics of the 978 Participants

Characteristic	No. (%)				*P* Value
	Total (N = 978)	Child Maltreatment History			
		None (n = 802)	Any (n = 176)		
Age, y^a^					
Mean (SD)	70.6 (2.9)	70.7 (2.9)		70.3 (2.8)	.046
Range	65-75	65-75		65-75	NA
Sex					
Men	426 (43.6)	335 (41.8)		91 (51.7)	.06
Women	540 (55.2)	457 (57.0)		83 (47.2)	
Missing	12 (1.2)	10 (1.2)		2 (1.1)	NA
Education, y					
<6	6 (0.6)	4 (0.5)		2 (1.1)	.01
6-9	232 (23.7)	175 (21.8)		57 (32.4)	
10-12	424 (43.4)	348 (43.4)		76 (43.2)	
≥13	307 (31.4)	266 (33.2)		41 (23.3)	
Other	2 (0.2)	2 (0.2)		0	
Missing	7 (0.7)	7 (0.9)		0	NA
Employment					
Working	183 (18.7)	146 (18.2)		37 (21.0)	.55
Retired	655 (67.0)	537 (67.0)		118 (67.0)	
Never employed	110 (11.2)	95 (11.8)		15 (8.5)	
Missing	30 (3.1)	24 (3.0)		6 (3.4)	NA
Longest-held occupation					
Specialist or technician	188 (19.2)	155 (19.3)		33 (18.8)	.16
Manager	66 (6.7)	56 (7.0)		10 (5.7)	
Clerical worker	204 (20.9)	172 (21.4)		32 (18.2)	
Sales or service job	208 (21.3)	166 (20.7)		42 (23.9)	
Manual labor	86 (8.8)	66 (8.2)		20 (11.4)	
Agriculture, forestry, or fisheries	8 (0.8)	7 (0.9)		1 (0.6)	
Self-employed	23 (2.4)	18 (2.2)		5 (2.8)	
Other	73 (7.5)	53 (6.6)		20 (11.4)	
Never had a job	51 (5.2)	47 (5.9)		4 (2.3)	
Missing	71 (7.3)	62 (7.7)		9 (5.1)	NA
Marital status					
Married	741 (75.8)	613 (76.4)		128 (72.7)	.27
Divorced	129 (13.2)	105 (13.1)		24 (13.6)	
Widowed	48 (4.9)	34 (4.2)		14 (8.0)	
Never married	44 (4.5)	35 (4.4)		9 (5.1)	
Other	7 (0.7)	7 (0.9)		0	
Missing	9 (0.9)	8 (1.0)		1 (0.6)	NA
Annual household income in millions, ¥^b^					
<1.5	146 (14.9)	114 (14.2)		32 (18.2)	.31
1.5-2.9	368 (37.6)	296 (36.9)		72 (40.9)	
3.0-4.9	260 (26.6)	218 (27.2)		42 (23.9)	
≥5.0	136 (13.9)	118 (14.7)		18 (10.2)	
Missing	68 (7.0)	56 (7.0)		12 (6.8)	NA
Living status					
Alone	153 (15.6)	117 (14.6)		36 (20.5)	.27
With family	767 (78.4)	638 (79.6)		129 (73.3)	
Other	15 (1.5)	12 (1.5)		3 (1.7)	
Missing	43 (4.4)	35 (4.4)		8 (4.5)	NA
Self-rated health					
Excellent	121 (12.4)	112 (14.0)		9 (5.1)	<.001
Good	686 (70.1)	567 (70.7)		119 (67.6)	
Fair	126 (12.9)	88 (11.0)		38 (21.6)	
Poor	15 (1.5)	10 (1.2)		5 (2.8)	
Missing	30 (3.1)	25 (3.1)		5 (2.8)	NA
Smoking					
Current	107 (10.9)	84 (10.5)		23 (13.1)	.15
Former	149 (15.2)	114 (14.2)		35 (19.9)	
No	714 (73.0)	597 (74.4)		117 (66.5)	
Missing	8 (0.8)	7 (0.9)		1 (0.6)	NA
Alcohol use					
Current	394 (40.3)	322 (40.1)		72 (40.9)	.98
Former	48 (4.9)	39 (4.9)		9 (5.1)	
No	527 (53.9)	434 (54.1)		93 (52.8)	
Missing	9 (0.9)	7 (0.9)		2 (1.1)	NA
Disease history					
High blood pressure	364 (37.2)	305 (38.0)		59 (33.5)	.26
Stroke	30 (3.1)	23 (2.9)		7 (4.0)	.44
Heart disease	83 (8.5)	70 (8.7)		13 (7.4)	.56
Diabetes	122 (12.5)	93 (11.6)		29 (16.5)	.08
Hyperlipidemia, ie, lipid abnormality	146 (14.9)	119 (14.8)		27 (15.3)	.87
Respiratory disease	43 (4.4)	34 (4.2)		9 (5.1)	.61
Gastrointestinal, liver, or gallbladder disease	77 (7.9)	59 (7.4)		18 (10.2)	.20
Kidney or prostate gland disease	57 (5.8)	38 (4.7)		19 (10.8)	.002
Musculoskeletal disease	99 (10.1)	74 (9.2)		25 (14.2)	.047
Traumatic injury	19 (1.9)	17 (2.1)		2 (1.1)	.39
Cancer	44 (4.5)	33 (4.1)		11 (6.3)	.22
Blood or immune system disease	14 (1.4)	13 (1.6)		1 (0.6)	.29
Depression	10 (1.0)	7 (0.9)		3 (1.7)	.32
Dementia	6 (0.6)	5 (0.6)		1 (0.6)	.93
Parkinson disease	3 (0.3)	3 (0.4)		0	.42
Eye disease	171 (17.5)	134 (16.7)		37 (21.0)	.17
Ear disease	51 (5.2)	39 (4.9)		12 (6.8)	.29
Other diseases	86 (8.8)	66 (8.2)		20 (11.4)	.18
**Prevalence of Childhood Maltreatment**					
Witnessing IPV					
Yes	44 (4.5)	NA		NA	NA
Missing	8 (0.8)	NA		NA	NA
Physical abuse					
Yes	19 (1.9)	NA		NA	NA
Missing	11 (1.1)	NA		NA	NA
Emotional neglect					
Yes	104 (10.6)	NA		NA	NA
Missing	16 (1.6)	NA		NA	NA
Emotional abuse					
Yes	56 (5.7)	NA		NA	NA
Missing	14 (1.4)	NA		NA	NA
Childhood maltreatment types, No.					
≥2	36 (3.7)	NA		NA	NA
1	140 (14.3)	NA		NA	NA
0	802 (82.0)	NA		NA	NA

Abbreviations: IPV, intimate partner violence; NA, not applicable.

^a^ Age was missing for 15 participants (1.5%).

^b^ Currency exchange rate is US$0.0092 per ¥1 (as of December 9, 2019).

As shown in Table 2, co-occurrence of childhood maltreatment was high among participants who experienced physical abuse. Of 19 participants who experienced physical abuse in childhood, 10 (52.6%) also experienced emotional neglect and 11 (57.9%) experienced emotional abuse. Witnessing IPV was more likely to happen independently from other childhood maltreatment (8 of 44 participants [18.2%] witnessed IPV with an additional experience of childhood maltreatment; 8 of 19 [42.1%] experienced physical abuse with an additional experience of childhood maltreatment; 23 of 104 [22.1%] experienced emotional neglect with an additional experience of childhood maltreatment; 19 of 56 [33.9%] experienced emotional abuse with an additional experience of childhood maltreatment).

**Table 2.** Co-occurrence of Childhood Maltreatment

Childhood Maltreatment	Sample Size, No.	No. (%)				
		Witness of IPV	Physical Abuse	Emotional Neglect	Emotional Abuse	Any 1 Additional Childhood Maltreatment
Witness of IPV	44	NA	5 (11.4)	10 (22.7)	5 (11.4)	8 (18.2)
Physical abuse	19	5 (26.3)	NA	10 (52.6)	11 (57.9)	8 (42.1)
Emotional neglect	104	10 (9.6)	10 (9.6)	NA	21 (20.2)	23 (22.1)
Emotional abuse	56	5 (8.9)	11 (19.6)	21 (37.5)	NA	19 (33.9)

Abbreviations: IPV, intimate partner violence; NA, not applicable.

Annual medical costs in FY2012 and FY2013 ranged from 0 to ¥5 201 690 (US$47 856) (mean, ¥437 569 [US$4026]; SD, ¥603 765 [US$5555]; median, ¥250 758 [US$2307]; interquartile range, ¥124 700-¥472 365 [US$1147-US$4346]). The distribution of mean annual medical costs is shown in the eFigure in the Supplement. This was similar to national mean medical costs; the mean sum of outpatient, inpatient, and pharmaceutical costs per person nationwide in FY2012 was ¥428 900 (US$3946) among adults aged 65 to 69 years and ¥565 900 (US$5206) among adults aged 70 to 74 years. In FY2013, it was ¥433 500 (US$3988) among adults aged 65 to 69 years and ¥576 600 (US$5305) among adults aged 70 to 74 years.^36,37^ Results from *t* tests showed that mean annual medical costs among older adults with any childhood maltreatment were ¥136 456 (US$1255) (95% CI, ¥38 155 to ¥234 757 [US$351 to US$2160]) higher than costs among those without childhood maltreatment (*P* = .007) (Table 3). Mean annual medical costs among those who experienced physical abuse and those who experienced emotional neglect were significantly higher than costs among their counterparts (physical abuse: difference, ¥295 148 [US$2715]; 95% CI, ¥206 631 to ¥569 693 [US$1901 to US$5241]; *P* = .04; emotional neglect: difference, ¥161 400 [US$1484]; 95% CI, ¥42 779 to ¥280 021 [US$394 to US$2576]; *P* = .008). Experiencing emotional abuse and witnessing IPV during childhood were not associated with medical costs in late adulthood (emotional abuse: difference, ¥56 649 ($521); 95% CI, −¥103 869 to ¥223 167 [−US$956 to US$2053]; *P* = .47; witnessing IPV: difference, −¥2604 [−US$24]; 95% CI, −¥186 186 to ¥180 979 [−US$1713 to US$1665]; *P* = .98).

**Table 3.** Unadjusted Annual Medical Costs With and Without History of Child Maltreatment, by Type of Childhood Maltreatment

History of Childhood Maltreatment	No.	Mean (SD), ¥^a^	Difference (95% CI), ¥^a^	*P* Value
**Any Childhood Abuse**				
Yes	176	549 468 (577 055)	136 456 (38 155 to 234 757)	.007
No	802	413 013 (704 002)		
**Witnessing IPV** ^**b**^				
Yes	44	435 855 (608 790)	–2604 (−186 186 to 180 979)	.98
No	926	438 459 (549 975)		
**Physical Abuse** ^**b**^				
Yes	19	726 254 (824 506)	295 148 (206 031 to 569 693)	.04
No	948	431 106 (598 811)		
**Emotional Neglect** ^**b**^				
Yes	104	573 481 (704 912)	161 400 (42 779 to 280 021)	.008
No	858	412 082 (565 609)		
**Emotional Abuse** ^**b**^				
Yes	56	493 650 (670 280)	59 649 (−103 869 to 223 167)	.47
No	908	434 001 (600 984)		
**Childhood Maltreatment Types, No.**				
≥2	36	509 464 (601 267)	96 452 (−110 575 to 303 479)	.35
1	140	559 755 (729 677)	146 742 (18 537 to 274 948)	.03
0	802	413 013 (577 055)	NA	NA

Abbreviations: IPV, intimate partner violence; NA, not applicable.

^a^ Currency exchange rate is US$0.0092 per ¥1 (as of December 9, 2019).

^b^ Missing values were excluded for *t *tests.

Mean annual medical costs among those who experienced 1 type of childhood maltreatment were significantly higher than costs among those who experienced none (difference, ¥96 452 [US$887]; 95% CI, −¥110 575 to ¥303 479 [−US$1017 to US$2792]; *P* = .03). Mean annual medical costs among those who experienced more than 2 types of childhood maltreatment were not significantly different from medical costs among those who experienced none.

The GLMs revealed that the association of any childhood maltreatment with annual medical costs remained statistically significant after controlling for age and sex in Model 1 (average marginal effect [AME], ¥116 098 [US$1068]; SE, ¥53 620 [US$493]; 95% CI, ¥11 004 to ¥221 192 [US$101 to US$2035]; *P* = .03) (Table 4). Based on these values, the prevalence of any childhood maltreatment in our sample (ie, 18.0%), and the total population of individuals aged 65 to 74 years in FY2012 to FY2013 in Japan (ie, 16 million),^38,39^ we can estimate that the additional annual medical costs associated with childhood maltreatment were more than ¥333 billion (US$3.1 billion) (95% CI, ¥32 billion to ¥637 billion [US$294.4 million to US$5.9 billion]) nationwide. When kidney or prostate gland disease and musculoskeletal disease were added in model 2, any childhood maltreatment was not associated with annual medical costs (AME, ¥101 214 [US$931]; SE, ¥53 961 [US$496]; 95% CI, –¥4548 to ¥206 977 [–US$42 to US$1904]; *P* = .06).

**Table 4.** Average Marginal Effects of Childhood Maltreatment on Annual Medical Costs

Adjustment	AME (SE) [95% CI]^a^	
	Model 1^b^	Model 2^c^
Any childhood abuse	116 098 (53 620) [11 004 to 221 192]	101 214 (53 961) [–4548 to 206 977]
Age	22 509 (6318) [10 126 to 34 892]	19 060 (6323) [6666 to 31 454]
Women	0 [Reference]	0 [Reference]
Men	163 405 (40 780) [83 477 to 243 332]	131 737 (40 082) [53 179 to 210 296]
Kidney or prostate gland disease	NA	346 100 (130 765) [89 806 to 602 394]
Musculoskeletal disease	NA	77 446 (52 332) [–25 123 to 180 016]
Witness of IPV^d^	4968 (72 119) [–136 382 to 146 318]	NA
Age	21 281 (6586) [8372 to 34 190]	NA
Women	0 [Reference]	NA
Men	179 378 (41 381) [98 273 to 260 483]	NA
Physical abuse	274 158 (210 099) [–137 629 to 685 946]	81 912 (149 570) [–211 240 to 375 063]
Age	22 696 (6370) [10 212 to 35 180]	21 449 (6326) [9051 to 33 847]
Women	0 [Reference]	0 [Reference]
Men	170 938 (41 384) [89 828 to 252 048]	148 027 (39 759) [70 101 to 225 953]
Stroke	NA	310 256 (147 798) [20 576 to 599 935]
Cancer	NA	655 566 (142 776) [375 730 to 935 402]
Emotional neglect	123 765 (66 083) [–5756 to 253 286]	108 215 (66 279) [–21 689 to 238 119]
Age	21 615 (6186) [9491 to 33 739]	19 367 (6181) [7252 to 31 481]
Women	0 [Reference]	0 [Reference]
Men	166 334 (39 587) [88 744 to 243 923]	138 589 (38 679) [62 779 to 214 399]
Kidney or prostate gland disease	NA	262 259 (117 263) [32 429 to 492 089]
Depression	NA	82 608 (88 717) [–91 274 to 256 491]
Emotional abuse^d^	54 540 (94 798) [–131 260 to 240 339]	NA
Age	22 102 (6423) [9514 to 34 690]	NA
Women	0 [Reference]	NA
Men	172 635 (41 571) [91 158 to 254 111]	NA
Childhood maltreatment types, No.		NA
0	0 [Reference]	NA
1	122 173 (58 373) [7763 to 236 582]	NA
≥2	92 150 (118 639) [–140378 to 324 677]	NA
Age	22 390 (6274) [10 093 to 34 687]	NA
Women	0 [Reference]	NA
Men	163 110 (40 684) [83 370 to 242 848]	NA

Abbreviations: AME, average marginal effect; IPV, intimate partner violence; NA, not applicable.

^a^ Calculated with delta method. Currency exchange rate is US$0.0092 per ¥1 (as of December 9, 2019).

^b^ Adjusted for age and sex.

^c^ Adjusted for older adults’ age, sex, and diseases associated with each type of childhood maltreatment.

^d^ Because *t *tests were not significant, model 2 was not run for this type of childhood maltreatment.

Emotional neglect was also not associated with mean annual medical costs (model 1: AME, ¥123 765 [US$1139]; SE, ¥66 083 [US$608]; 95% CI, −¥5756 to ¥253 286 [−US$53 to US$2330]; *P* = .06; model 2: AME, ¥108 215 [US$996]; SE, ¥66 279 [US$610]; 95% CI, −¥21 689 to ¥238 119 [−US$200 to US$2191]; *P* = .10). Mean annual medical costs among those with childhood physical abuse were not significantly different than costs among those without childhood physical abuse, although the difference was largest in model 1 (model 1: AME, ¥274 158 [US$2522]; SE, ¥210 009 [US$1932]; 95% CI, –¥137 629 to ¥685 946 [–US$1266 to US$6311]; *P* = .19; model 2: AME, ¥81 912 [US$754]; SE, ¥149 570 [US$1376]; 95% CI, –¥211 240 to ¥375 063 [–US$1943 to US$3451]; *P* = .58). Similar to results from *t* tests (Table 3), mean annual medical costs among those with emotional abuse and those who witnessed IPV did not significantly differ from costs among their counterparts in model 1 (emotional abuse: AME, ¥54 540 [US$502]; SE, ¥94 798 [US$872]; 95% CI, –¥131 260 to ¥240 339 [–US$1208 to US$2211]; *P* = .57; witness of IPV: AME, ¥4968 [US$46]; SE, ¥72 119 [US$663]; 95% CI, –¥136 382 to ¥146 318 [–US$1255 to US$1346]; *P* = .95).

Finally, the association of total number of childhood maltreatment types with mean annual medical costs among older adults was also examined in the adjusted model. Those who experienced 1 type of childhood maltreatment had significantly higher total annual medical costs than those who experienced none (AME, ¥122 173 [US$1124]; SE, ¥58 373 [US$537]; 95% CI, ¥7763-¥236 582 [US$71-US$2177]; *P* = .04). However, mean annual medical costs among those who experienced more than 2 types of childhood maltreatment did not significantly differ from medical costs among those who experienced none.

## Discussion

To our knowledge, this is the first study that examined additional annual medical costs associated with childhood maltreatment among independent older adults aged 65 to 74 years. Using the database of health insurance claims that is linked with data from a population-based cohort study in Japan, we found that mean annual medical costs among those with any history of childhood maltreatment were significantly higher than among those with no history of maltreatment.

Older adults with a history of childhood physical abuse had ¥295 148 higher medical costs than those without; however, the increase was not statistically significant when their age and sex were considered. This was not consistent with a previous study,^30^ which reported that total annual health care costs calculated from health care system records were significantly higher among women with a history of physical abuse compared with women without, even after adjusting for calendar year, age, and education. However, the previous study only included women aged 18 to 64 years, while the target population of our study was men and women aged 65 to 74 years. Although we had smaller sample size, our study examined longer-term consequences of physical abuse among both women and men than previous studies. Findings on the other types of maltreatment cannot be compared directly with previous studies because, to our knowledge, no studies have investigated the long-term associations of witnessing IPV or experiencing emotional neglect and abuse in childhood with medical costs.

Having more than 1 type of childhood maltreatment or adverse childhood experience has been reported to increase the risk of health problems^1,4,18,20,40,41,42,43^ and health care costs later in life.^28,30,31^ However, our study found no significant difference in the long-term medical costs between those with more than 2 types of maltreatment and those with no history of child maltreatment. This can be explained by a selective survival effect,^44,45^ ie, those with a history of multiple childhood maltreatment types were more likely to have been excluded from our sample because they may have died or become functionally dependent before they reached the target age of our study (ie, 65-74 years), given that having more adverse childhood experiences has been found to be associated with premature mortality.^25,46,47^

Our study also explored whether disease history mediated the association of childhood maltreatment with medical costs in older adulthood. Our findings are consistent with previous studies, in that the long-term association of child maltreatment with health care costs seemed to be mediated by physical and mental health concerns.^31^ The magnitude of the association of childhood maltreatment with medical costs was largely reduced when diseases associated with childhood maltreatment were added to our analysis model. Furthermore, our study implied that childhood physical abuse was associated with an increased risk of stroke and cancer, while emotional neglect was associated with an increased risk of kidney or prostate gland disease, which may have led to additional medical costs among independent older adults aged 65 to 74 years. This finding is in line with a previous study using the 2012 Canadian Community Health Survey data that indicated childhood physical abuse was associated with stroke, cancer, and other diseases.^48^ Emotional neglect has also been found to be associated with an increased risk of such diseases,^49^ but its association with kidney or prostate gland diseases had not been found in previous studies. Because there are a limited number of studies that examine the association of specific types of child maltreatment, especially emotional neglect, with adult diseases and different measures of diseases were used in these studies, future research using objective measures of diseases, including a wide range of diseases associated with child maltreatment, such as substance use disorders and mood disorders, is warranted to elucidate what kinds of diseases could explain the association of childhood maltreatment with increased medical costs in later life.

Furthermore, we estimated that additional medical costs associated with childhood maltreatment were more than ¥333 billion (US$3.1 billion) per year nationwide, which is approximately 0.9% of total annual medical spending (ie, the sum of outpatient, inpatient, and pharmaceutical costs) in Japan in FY2012 and FY2013 and 8.4% of total annual medical spending among those aged 65 to 74 years in FY2012 and FY2013.^36,37^ These figures suggest tremendous long-term consequences of childhood maltreatment, considering that this estimate is only for those aged 65 to 74 years who are functionally independent. Additionally, the estimate from this study is more than twice that of indirect medical costs (¥136 billion [US$1.3 billion]) estimated in the previous study in Japan.^32^ Our findings could contribute to more accurate estimations of the costs of child maltreatment using real health insurance claims data in Japan.

### Limitations

There are several limitations of this study. First, we might have overexcluded those who did not use national health insurance (ie, those whose medical costs were ¥0) because we could not completely distinguish between participants who were eligible for national health insurance but did not use it and those who were not eligible for national health insurance. However, a comparison of demographic information between those whose medical costs in both FY2012 and FY2013 were not available and those whose medical costs were available implies that those who were excluded from the sample seemed likely to be ineligible for national health insurance (eTable 2 in the Supplement). In addition, the prevalence of childhood maltreatment was not significantly different between these groups (eTable 2 in the Supplement), and similar results on the association between childhood maltreatment and medical costs were obtained from GLM analysis including those whose medical costs were ¥0 (data not shown). Second, retrospective self-reports of childhood maltreatment were used in our study, which can be subject to recall bias.^50,51^ Given that another study^52^ suggested retrospective measures are more likely to produce false-negatives than false-positives, our findings on the association of childhood maltreatment with medical costs may be underestimated. Third, childhood sexual abuse was not assessed in our study because of the feasibility of the survey in the cultural context of Japan. However, a population-based study in Japan reported that the prevalence of childhood sexual abuse was quite low (ie, 0.5%),^53^ which contributed little to the underestimation of prevalence of childhood maltreatment. Furthermore, even if childhood sexual abuse were queried in surveys, previous studies in Japan^54,55^ have reported low response rates (ie, 19.1% and 25.6%, respectively). Nonetheless, future research needs to consider how to assess childhood sexual abuse and its long-term association with health and health care costs in Japan, considering that childhood sexual abuse has been found to have significant consequences on health and health care costs later in life in other countries.^28,29,30,31^ Fourth, survival selection bias could underestimate the association of childhood maltreatment with medical costs. Fifth, the generalizability of our findings may be limited because our study only included independent adults aged 65 to 74 years in a particular city. When available, future research should include such older adults to better understand the long-term association of childhood maltreatment with medical costs using a representative sample.

## Conclusions

Our study provided evidence that history of childhood maltreatment, particularly emotional neglect, was associated with increases in medical costs among older men and women. The findings demonstrated significant long-term consequences of childhood maltreatment and underlined the importance of primary prevention (ie, home visitation programs during pregnancy for high-risk populations) and early intervention (ie, screening of adverse childhood events and intervention in pediatric clinics) for child maltreatment.

## Notes

Supplement. **eFigure. **Histogram of Mean Annual Medical Costs **eTable 1. **Health-Related Behaviors and Health Conditions Associated With Each Type of Childhood Maltreatment **eTable 2. **Comparison of Characteristics and Prevalence of Childhood Maltreatment Depending on Availability of Medical Cost Data
