# Errors in Currency Conversion

**DOI:** 10.1001/jamanetworkopen.2020.0381

**Published:** 2020-02-05

In the Original Investigation titled “Assessment of Additional Medical Costs Among Older Adults in Japan With a History of Childhood Maltreatment,”^1^ published January 8, 2020, there were errors in the conversion from yen to dollars in the Abstract, Introduction, Results, and Discussion. This article has been corrected.^1^
