# Supplementary material for: Assessment of Additional Medical Costs Among Older Adults in Japan With a History of Childhood Maltreatment
Source: JAMA Netw Open. 2020 Jan 8;3(1):e1918681. doi: 10.1001/jamanetworkopen.2019.18681 (PMC6991253; doi:10.1001/jamanetworkopen.2019.18681)
Supplement: Supplement. — eFigure. Histogram of Mean Annual Medical Costs eTable 1. Health-Related Behaviors and Health Conditions Associated With Each Type of Childhood Maltreatment eTable 2. Comparison of Characteristics and Prevalence of Childhood Maltreatment Depending on Availability of Medical Cost Data [file jamanetwopen-e1918681-s001.pdf]

Supplementary Online Content

Isumi A, Fujiwara T, Kato H, et al. Assessment of additional medical costs among older adults in Japan with a history of childhood maltreatment. *JAMA Netw Open*. 2020;3(1):e1918681. doi:10.1001/jamanetworkopen.2019.18681

eFigure. Histogram of Mean Annual Medical Costs
eTable 1. Health-Related Behaviors and Health Conditions Associated With Each Type of Childhood Maltreatment
eTable 2. Comparison of Characteristics and Prevalence of Childhood Maltreatment Depending on Availability of Medical Cost Data

This supplementary material has been provided by the authors to give readers additional information about their work.

© 2020 Isumi A et al. *JAMA Network Open.*

eFigure. Histogram of Mean Annual Medical Costs

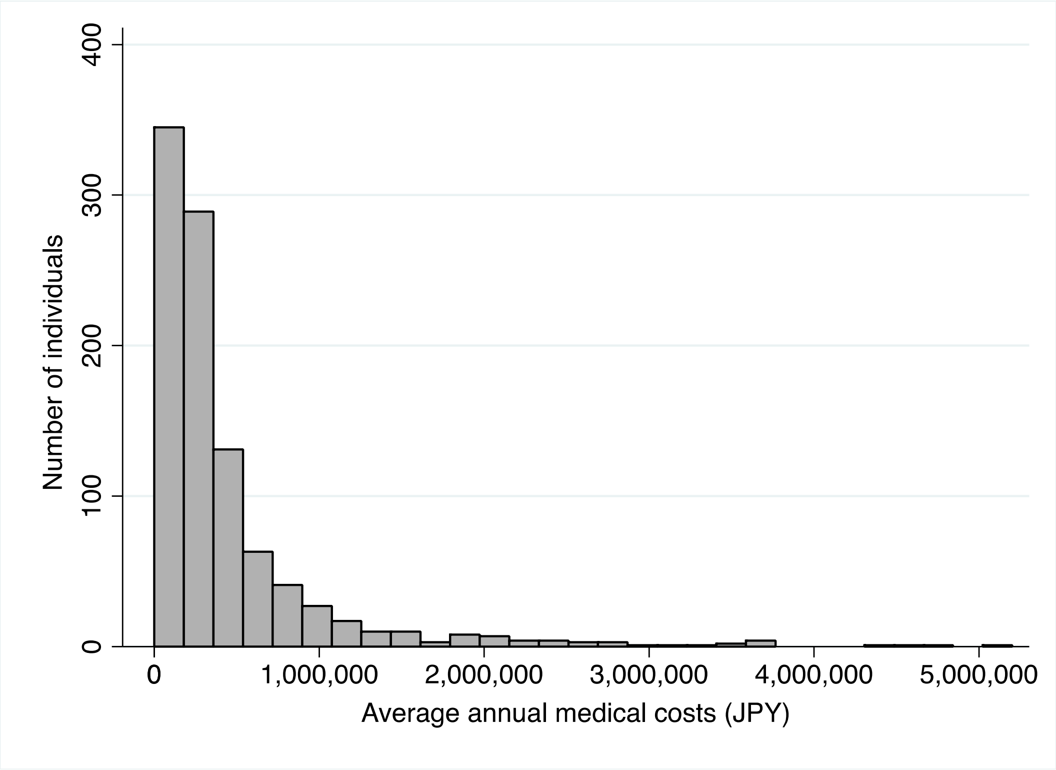

© 2020 Isumi A et al. *JAMA Network Open.*

eTable 1. Health-Related Behaviors and Health Conditions Associated With Each Type of Childhood Maltreatment

		Witness of IPV					Physical abuse					Emotional neglect					Emotional abuse				
		No (N=926)		Yes (N=44)			No (N=948)		Yes (N=19)			No (N=858)		Yes (N=104)			No (N=908)		Yes (N=56)		
**Health conditions**		**N**	**%**	**N**	**%**	***p***	**N**	**%**	**N**	**%**	***p***	**N**	**%**	**N**	**%**	***p***	**N**	**%**	**N**	**%**	***p***
Self-rated health	Excellent	115	12.8	3	7.0	0.191	118	12.9	0	0	0.011	117	14.1	3	3.0	<0.001	114	13	4	7.4	0.620
	Good	652	72.7	30	69.8		668	72.8	12	63.2		605	72.8	68	67.3		637	72.4	40	74.1	
	Fair	117	13.0	8	18.6		117	12.7	7	36.8		96	11.6	28	27.7		115	13.1	9	16.7	
	Poor	13	1.4	2	4.7		15	1.6	0	0		13	1.6	2	2.0		14	1.6	1	1.9	
Smoking	Yes	103	11.2	4	9.1	0.845	104	11.1	3	15.8	0.274	88	10.3	17	16.5	0.002	100	11.1	7	12.7	0.836
	Used to smoke	141	15.4	6	13.6		141	15.0	5	26.3		120	14.1	25	24.3		138	15.3	7	12.7	
	No	674	73.4	34	77.3		695	73.9	11	57.9		643	75.6	61	59.2		663	73.6	41	74.5	
Alcohol	Yes	374	40.7	17	38.6	0.416	380	40.4	11	57.9	0.101	343	40.4	44	42.7	0.888	366	40.6	22	40.7	0.963
	Used to drink	43	4.7	4	9.1		44	4.7	2	10.5		40	4.7	5	4.9		43	4.8	3	5.6	
	No	501	54.6	23	52.3		516	54.9	6	31.6		467	54.9	54	52.4		493	54.7	29	53.7	
Diseases history																					
High blood pressure	Yes	346	37.4	13	29.5	0.294	350	36.9	8	42.1	0.643	318	37.1	38	36.5	0.917	336	37	21	37.5	0.941
Stroke	Yes	28	3.0	2	4.5	0.569	27	2.8	3	15.8	0.001	25	2.9	5	4.8	0.294	27	3	3	5.4	0.319
Heart disease	Yes	77	8.3	3	6.8	0.724	77	8.1	2	10.5	0.705	72	8.4	8	7.7	0.807	76	8.4	4	7.1	0.747

© 2020 Isumi A et al. *JAMA Network Open.*

Diabetes	Yes	115	12.4	5	11.4	0.835	117	12.3	2	10.5	0.811	103	12.0	17	16.3	0.205	107	11.8	12	21.4	0.033
Hyperlipidemia (lipid abnormality)	Yes	136	14.7	9	20.5	0.294	140	14.8	4	21.1	0.446	127	14.8	15	14.4	0.918	137	15.1	7	12.5	0.598
Respiratory disease	Yes	37	4.0	4	9.1	0.101	38	4.0	2	10.5	0.158	38	4.4	4	3.8	0.784	41	4.5	0	0	0.104
Gastrointestinal, liver, or gallbladder disease	Yes	74	8.0	3	6.8	0.779	75	7.9	1	5.3	0.671	66	7.7	10	9.6	0.492	70	7.7	6	10.7	0.418
Kidney or prostate gland disease	Yes	53	5.7	4	9.1	0.353	54	5.7	2	10.5	0.372	43	5.0	11	10.6	0.020	51	5.6	5	8.9	0.304
Musculoskeletal disease	Yes	88	9.5	10	22.7	0.004	96	10.1	2	10.5	0.954	83	9.7	13	12.5	0.364	92	10.1	5	8.9	0.771
Traumatic injury	Yes	17	1.8	2	4.5	0.205	19	2.0	0	0	0.533	18	2.1	0	0	0.136	18	2	0	0	0.288
Cancer	Yes	39	4.2	5	11.4	0.026	40	4.2	3	15.8	0.015	37	4.3	4	3.8	0.824	39	4.3	4	7.1	0.316
Blood or immune system disease	Yes	14	1.5	0	0	0.411	14	1.5	0	0	0.594	13	1.5	1	1.0	0.656	13	1.4	1	1.8	0.830
Depression	Yes	10	1.1	0	0	0.488	10	1.1	0	0	0.653	7	0.8	3	2.9	0.049	10	1.1	0	0	0.430
Dementia	Yes	6	0.6	0	0	0.592	6	0.6	0	0	0.728	5	0.6	1	1.0	0.643	6	0.7	0	0	0.542
Parkinson's disease	Yes	3	0.3	0	0	0.705	3	0.3	0	0	0.806	3	0.3	0	0	0.546	3	0.3	0	0	0.667
Eye disease	Yes	159	17.2	10	22.7	0.342	161	17.0	6	31.6	0.096	148	17.2	19	18.3	0.795	150	16.5	16	28.6	0.020
Ear disease	Yes	47	5.1	3	6.8	0.610	49	5.2	1	5.3	0.985	43	5.0	6	5.8	0.740	45	5	5	8.9	0.193
Other diseases	Yes	84	9.1	2	4.5	0.302	83	8.8	3	15.8	0.286	72	8.4	12	11.5	0.283	77	8.5	8	14.3	0.137

© 2020 Isumi A et al. *JAMA Network Open.*

eTable 2. Comparison of Characteristics and Prevalence of Childhood Maltreatment Depending on Availability of Medical Cost Data

		FY2012/2013 Medical cost data				
		Not available (N=2,102)		Available (N=5,155)		
**Characteristics of the sample**		**N or Mean**	**% or SD**	**N or Mean**	**% or SD**	***p***
Age	65-75	69.0	3.2	70.5	2.9	<0.001
Sex	Men	1055	50.2	2237	43.4	<0.001
	Women	963	45.8	2842	55.1	
	Missing	84	4.0	76	1.5	
Education	< 6 years	28	1.3	20	0.4	<0.001
	6−9 years	518	24.6	1207	23.4	
	10−12 years	784	37.3	2292	44.5	
	≥13 years	668	31.8	1526	29.6	
	Others	12	0.6	21	0.4	
	Missing	92	4.4	89	1.7	
Employment	Working	788	37.5	1011	19.6	<0.001
	Retired	943	44.9	3268	63.4	
	Never employed	175	8.3	542	10.5	
	Missing	196	9.3	334	6.5	
Occupation (Longest)	Specialist, technician	372	17.7	881	17.1	<0.001

© 2020 Isumi A et al. *JAMA Network Open.*

	Manager	213	10.1	345	6.7	
	Clerical worker	346	16.5	1075	20.9	
	Sales/service jobs	391	18.6	1049	20.3	
	Manual labor	202	9.6	466	9.0	
	Agriculture, forestry or fisheries	7	0.3	39	0.8	
	Self-employed	76	3.6	151	2.9	
	Other	184	8.8	401	7.8	
	Never had a job	64	3.0	228	4.4	
	Missing	247	11.8	520	10.1	
Marital status	Married	1380	65.7	3826	74.2	<0.001
	Divorced	282	13.4	717	13.9	
	Widowed	191	9.1	237	4.6	
	Never married	119	5.7	234	4.5	
	Other	38	1.8	45	0.9	
	Missing	92	4.4	96	1.9	
Annual household income	<1.5 million	350	16.7	783	15.2	<0.001
	1.5−2.9 million	477	22.7	1907	37.0	
	3.0−4.9 million	478	22.7	1284	24.9	
	≥5.0 million	488	23.2	658	12.8	
	Missing	309	14.7	523	10.1	
Living status	Living alone	457	21.7	842	16.3	<0.001
	Living with family	1494	71.1	3999	77.6	

© 2020 Isumi A et al. *JAMA Network Open.*

	Other	31	1.5	67	1.3	
	Missing	120	5.7	247	4.8	
**Prevalence of childhood maltreatment**						
Witnessing DV	No	384	93.2	926	94.7	0.189
	Yes	20	4.9	44	4.5	
	Missing	8	1.9	8	0.8	
Physical abuse	No	395	95.9	948	96.9	0.465
	Yes	9	2.2	19	1.9	
	Missing	8	1.9	11	1.1	
Emotional neglect	No	361	87.6	858	87.7	0.184
	Yes	49	11.9	104	10.6	
	Missing	2	0.5	16	1.6	
Emotional abuse	No	378	91.7	908	92.8	0.711
	Yes	26	6.3	56	5.7	
	Missing	8	1.9	14	1.4	

© 2020 Isumi A et al. *JAMA Network Open.*
